# Serum Levels of 54 Cytokines and Chemokines Reveal Distinct Inflammatory Signatures in Ankylosing Spondylitis

**DOI:** 10.1002/iid3.70276

**Published:** 2025-10-15

**Authors:** Huan Li, Ting Wang, Mingze Li, Wei Su, Ying Lv, Jialing Xiao, Xiaoxin Guo, Kai Dong, Chengzi Gan, Jing Zhu, Bo Gong

**Affiliations:** ^1^ Department of Health Management, Sichuan Academy of Medical Sciences & Sichuan Provincial People's Hospital School of Medicine, University of Electronic Science and Technology of China Chengdu Sichuan China; ^2^ Human Disease Genes Key Laboratory of Sichuan Province and Institute of Laboratory Medicine, Sichuan Academy of Medical Sciences & Sichuan Provincial People's Hospital University of Electronic Science and Technology of China Chengdu Sichuan China; ^3^ Research Unit for Blindness Prevention of Chinese Academy of Medical Sciences (2019RU026), Sichuan Academy of Medical Sciences & Sichuan Provincial People's Hospital University of Electronic Science and Technology of China Chengdu Sichuan China; ^4^ Department of Rheumatology and Immunology, Sichuan Academy of Medical Sciences & Sichuan Provincial People's Hospital University of Electronic Science and Technology of China Chengdu Sichuan China

**Keywords:** ankylosing spondylitis, chemokines, cytokines

## Abstract

**Background:**

Ankylosing spondylitis (AS) is a chronic autoimmune inflammatory disorder predominantly involving the axial skeleton. Understanding the cytokine and chemokine signatures in AS is crucial for elucidating disease mechanisms and identifying potential diagnostic biomarkers.

**Methods:**

Serum samples from 31 AS patients and 20 age‐matched healthy controls (HCs) were analyzed. The concentrations of 54 cytokines, chemokines, and angiogenesis‐related factors were measured using a Meso Scale Discovery (MSD) electrochemiluminescence immunoassay. Data analysis included statistical comparison of serum cytokine levels, heatmap clustering, principal component analysis (PCA), and receiver operating characteristic (ROC) curve analysis. Validation was performed using peripheral blood mononuclear cells (PBMCs) from SKG mice, a spontaneous animal model of AS, through quantitative real‐time PCR.

**Results:**

Compared with HCs, AS patients showed significantly higher serum concentrations of 12 cytokines (TNF‐α, TNF‐β, IL‐17A, IL‐17D, VEGFA, ICAM1, SAA, IP‐10/CXCL10, MIP‐3α/CCL20, sFlt‐1/VEGFR‐1, CRP, and MCP‐4/CCL13) and significantly lower concentrations of nine cytokines (IL‐4, IL‐8, IL‐17C, MIP‐1α/CCL3, eotaxin‐3/CCL26, PlGF, VEGF‐C, VEGF‐D, and bFGF) (all *p* < 0.05). Heatmap clustering and PCA demonstrated a clear separation between AS patients and HCs. ROC curve analysis showed excellent diagnostic accuracy for IP‐10/CXCL10 (AUC = 1.00), VEGF‐D (AUC = 0.98), IL‐17A (AUC = 0.87), TNF‐α (AUC = 0.85), and ICAM1 (AUC = 0.84). Positive correlations were observed between IL‐17A and MIP‐3α/CCL20, and between VEGFA and sFlt‐1, indicating coordinated inflammatory and angiogenic pathways. Validation experiments in SKG mice confirmed elevated IP‐10/CXCL10 and reduced VEGF‐D expression, supporting cross‐species relevance.

**Conclusions:**

This study identified distinct cytokine and chemokine profiles in AS patients. IP‐10/CXCL10 and VEGF‐D emerged as promising diagnostic biomarkers with high discriminatory power. Several previously unreported immune mediators were also highlighted. These findings provide new insights into AS pathogenesis and suggest potential targets for future therapeutic interventions.

## 1 Introduction

Ankylosing spondylitis (AS) refers to a chronic inflammatory rheumatic condition mostly involving the axial skeleton and is characterized by inflammatory back pain, sacroiliitis, progressive spinal ankylosis, and enthesitis [1]. Epidemiological studies have reported that the prevalence of AS varies globally, with about 0.1%–1.4% affected, indicating notable regional and ethnic differences [2, 3]. The disease generally emerges in individuals in their thirties, with its highest incidence occurring between the ages of 30 and 39, leading to significant economic and healthcare burdens [4, 5].

Even though the specific pathogenic mechanism of AS remains unknown, many researchers have reported that this disease occurs due to complicated interactions between genetic and environmental factors, which, in turn, activate immune‐mediated inflammatory pathways that drive its onset and progression [6]. Among genetic factors, HLA‐B27 is the strongest known risk factor for AS, with over 90% of AS patients carrying this allele [7, 8]. However, the precise mechanisms by which HLA‐B27 influences AS pathogenesis remain incompletely understood. Immune dysregulation involving abnormal cytokine and chemokine responses plays a key role in driving chronic inflammation, joint damage, and disease progression in AS [9, 10].

Cytokines and chemokines make vital impacts on regulating immune responses by mediating the recruitment, activation, and differentiation of leukocytes and sustaining inflammation. Aberrant expression of inflammatory factors occurs in different autoimmune and rheumatic disorders, including rheumatoid arthritis (RA), systemic lupus erythematosus (SLE), and psoriatic arthritis (PsA) [11, 12, 13]. Similarly, the cytokine profiles in AS patients are altered, although comprehensive characterization and understanding of these patterns remain limited [14, 15]. Previous studies focused primarily on well‐known inflammatory cytokines, such as tumor necrosis factor‐alpha (TNF‐α), interleukin‐17 (IL‐17), and IL‐23, which play important roles in the pathogenesis of AS and are therapeutic targets of biologic agents [16, 17, 18]. However, the involvement of many other cytokines, chemokines, and angiogenic factors in AS remains poorly evaluated. Given the established connections between cytokine and chemokine dysregulation and inflammatory disorders, further assessment of these immune mediators may help elucidate the pathogenesis underlying AS.

In this study, we preliminarily analyzed and compared the serum profiles of cytokines, chemokines, and angiogenic mediators in AS patients and healthy controls (HCs). We focused on inflammatory cytokines and chemokines, including TNF‐α, TNF‐β, IL‐17A, IL‐17D, IL‐4, IL‐8, IP‐10/CXCL10, MIP‐3α/CCL20, MCP‐4/CCL13, VEGF family members, ICAM‐1, and other key inflammatory mediators, because of their established or potential involvement in the immunopathogenesis of AS. Additionally, bioinformatic analyses were conducted to elucidate potential functional relationships among these differentially expressed cytokines to reveal new diagnostic biomarkers and therapeutic targets of AS. However, the clinical specificity of these cytokines for differentiating between AS and other inflammatory conditions has not been evaluated. Further studies comparing cytokine signatures across multiple inflammatory diseases and assessing combined cytokine panels against the current gold standard for diagnosing AS are needed to validate their clinical applicability.

## 2 Materials and Methods

### 2.1 Human Samples

This study was approved by the Institutional Review Boards of Sichuan Provincial People's Hospital. A total of 31 AS patients and 20 HCs were enrolled between April 2018 and July 2022. All participants offered informed consent following the Declaration of Helsinki. None of the AS patients had received systemic immunosuppressive therapy, corticosteroids, or biologics for 1 week or longer before blood collection. Participants with a history of malignancies or autoimmune diseases were excluded. HCs were selected based on the absence of AS and known autoimmune disorders. Demographic and clinical data, including age, sex, the HLA‐B27 status, disease course, biologic therapy, Bath Ankylosing Spondylitis Disease Activity Index (BASDAI) score, and blood cell counts (neutrophils, leukocytes, lymphocytes, eosinophils, monocytes, and basophils), were collected.

### 2.2 Animal Study

Female SKG mice aged 6–8 weeks and age‐matched wild‐type BALB/c mice (WT controls), obtained from Shanghai Model Organisms Center Inc. (China), were housed under specific pathogen‐free (SPF) conditions with a controlled 12‐h light/dark cycle and constant temperature. Mice were maintained under SPF conditions with a controlled temperature (22 ± 2°C) and humidity (50%–60%). BALB/c mice served as the control group for SKG mice, and each group contained six mice (*n* = 6 per group). All experimental protocols in this study were approved by the Animal Care and Use Committee of Sichuan Provincial People's Hospital (approval number: A22‐15).

### 2.3 Sampling and Determination of Cytokines/Chemokines

Venous blood samples were obtained from every AS patient and HC using ethylenediaminetetraacetic acid (EDTA) tubes. The samples were left undisturbed for 2 h at ambient temperature before centrifugation at 3000 rpm for 10 min. Following careful separation, the serum samples were preserved before subsequent analyses. All serum samples were stored at –80°C and thawed only once before analysis to ensure stability.

Serum levels of cytokines and chemokines were measured by the Meso Scale Discovery (MSD) electrochemiluminescence platform using a V‐PLEX Human Cytokine 30‐Plex Kit following specific protocols. Briefly, after dilution with a buffer, plasma samples were placed at the bottom of duplicate wells in an assay plate. After incubation with detection antibodies and multiple washing steps, read buffer was applied, and the plates were analyzed using the MSD QuickPlex SQ120 instrument. The emitted light intensity was recorded to provide a quantitative assessment of the analytes. Each cytokine/chemokine assay was performed in duplicate, and the mean values were used for analysis. A total of 54 immune mediators were simultaneously assessed, which allowed a comprehensive inflammatory profile analysis.

The pro‐inflammatory cytokines and immune modulators analyzed included IFN‐γ, IL‐1β, IL‐2, IL‐4, IL‐6, IL‐8, IL‐10, IL‐12p70, IL‐13, TNF‐α, GM‐CSF, IL‐1α, IL‐5, IL‐7, IL‐12/IL‐23p40, IL‐15, IL‐16, IL‐17A, TNF‐β, VEGF‐A, IL‐17A/F, IL‐17B, IL‐17C, IL‐17D, IL‐1RA, IL‐3, IL‐9, and TSLP. The Th17‐related cytokines IL‐17A (Gen. B), IL‐21, IL‐22, IL‐23, IL‐27, and IL‐31 were also quantified. Chemokines included MIP‐3α/CCL20, Eotaxin/CCL11, MIP‐1β/CCL4, Eotaxin‐3/CCL26, TARC/CCL17, IP‐10/CXCL10, MIP‐1α/CCL3, MCP‐1/CCL2, MDC/CCL22, and MCP‐4/CCL13. The angiogenesis‐related mediators VEGF‐A, VEGF, VEGF‐C, VEGF‐D, Tie‐2, sFlt‐1/VEGFR‐1, PlGF, and bFGF were assessed. Markers of vascular injury included SAA, CRP, VCAM‐1, and ICAM‐1. The samples were explored in duplicate, and the results are indicated as the mean values of the two measurements. The coefficient of variation and the sensitivity for each analyte were within the manufacturer's expected range. Limits of detection for each analyte complied with the manufacturer's specifications.

### 2.4 Preparation of Single‐Cell Suspensions of SKG Mouse PMBC Samples

Fresh venous blood from SKG mice was gathered in EDTA‐coated anticoagulant tubes. The blood was later diluted using sterile phosphate‐buffered saline (PBS) at a ratio of 1:1. Equal volumes of diluted blood and Ficoll lymphocyte separation medium were gently layered in 50‐mL centrifuge tubes. Then, the samples were exposed to centrifugation at 2000 rpm and 20°C for 20 min using a horizontal rotor, with the brake set to zero to preserve cell layering. After carefully collecting the peripheral blood mononuclear cell (PBMC) layer, it was added to a 15‐mL tube. The cells were washed with 10 mL of PBS before centrifugation (300 × *g*, 10 min). The supernatants were removed, while the pellet was resuspended in 5 mL of PBS and centrifuged again under the same conditions. After two washing steps, the remaining cell pellet was gently resuspended in RPMI‐1640 medium (1 mL) supplemented with 0.04% BSA. The resulting single‐cell suspension was quantified using a Luna cell counter, and cell viability was assessed via Trypan blue exclusion.

### 2.5 qPCR

A Rotor‐Gene Q system (Qiagen, Germantown, Maryland, the United States) was used for Quantitative real‐time polymerase chain reaction (qPCR). Each 20 µL reaction mixture consisted of 10 µL of SYBR Green Master Mix (TransGen Biotech, Beijing), 1 µL of cDNA template (100 ng), and gene‐specific primers. Amplification was conducted over 45 cycles for all samples. Gene expression levels were quantified via the 2^−ΔΔCt^ approach. Following the MIQE guidelines, the experiments were conducted. The housekeeping gene 18S rRNA served as the internal control for normalization. All primers were synthesized by Sangon Biotech. The sequences and corresponding catalog numbers were as follows: IP‐10/CXCL10 (forward: CCAAGTGCTGCCGTCATTTTC; reverse: GGCTCGCAGGGATGATTTCAA) and VEGF‐D (forward: TTGAGCGATCATCCCGGTC; reverse: GCGTGAGTCCATACTGGCAAG).

### 2.6 Statistical Analysis

IBM SPSS Statistics 20 (IBM Corp, Armonk, New York, the United States) and GraphPad Prism 8 (GraphPad Software, San Diego, California, the United States) were adopted for data analysis. Outliers were removed before statistical testing. Data distribution was tested for normality, and either parametric (*t*‐test) or non‐parametric (Mann–Whitney *U*‐test) tests were applied accordingly. Cytokine and chemokine concentrations were compared between AS patients and HCs by conducting the Mann–Whitney *U*‐test and the unpaired *t*‐test. Using the Bonferroni correction, multiple comparisons via the Mann–Whitney *U*‐test were adjusted. Later, the data matrix was imported into the R package software to conduct multivariate statistical analyses, consisting of principal component analysis (PCA) (R package “prcomp”), heatmaps (R package “pheatmap”), and receiver operating characteristic (ROC) curves (R package “pROC”). Spearman's correlation test was employed to evaluate the correlations of cytokine levels with clinical parameters. AUC values were interpreted according to established standards. Demographic and clinical variables, including age, sex, HLA‐B27 status, biologic therapy, course of disease, and blood cell counts, were represented as the mean ± standard deviation (SD). All results were considered to be statistically significant at *p* < 0.05.

## 3 Results

### 3.1 Baseline Features of the Participants

A total of 31 AS patients and 20 HCs were involved in the present study. All participants satisfied the eligibility criteria. The demographic and clinical data for both groups are presented in Table 1. Age and sex distributions were comparable between AS patients and HCs. There were 74% and 75% males in the AS and HC groups, respectively, with average ages of 35.17 ± 10.76 years and 33.15 ± 8.96 years in these two groups. The average course of disease in the AS group was 6.22 years. All AS patients (100%) tested positive for HLA‐B27. Among the AS patients, 16 (51.6%) had previously received biologic therapy. The mean BASDAI score in the AS group was 3.82 ± 1.17. Compared to those in the HCs, the leukocyte, neutrophil, and monocyte counts in the AS group were significantly greater (*p* < 0.05), whereas the eosinophil and basophil counts were of no significant difference between the groups (*p* > 0.05).

**Table 1:** General clinical data in the AS and HCs groups.

	AS group (*n* = 31)	HCs group (*n* = 20)	Reference range	*p* value
Age (years)	35.17 ± 10.76	33.15 ± 8.96	NA	> 0.05
Sex (male/female)	23/8	15/5	NA	> 0.05
Disease duration	6.22 ± 4.81	NA	NA	NA
HLA‐B27 positive	31/31	NA	NA	NA
Biologic therapy	16/31	N/A	N/A	N/A
BASDAI	3.82 ± 1.17	N/A	N/A	N/A
Leukocytes (× 10^9^/L)	8.45 ± 1.44	4.89 ± 1.23	4.0–10.0	< 0.05
Neutrophils (× 10^9^/L)	5.47 ± 2.01	3.31 ± 1.03	2.5–7.5	< 0.05
Lymphocytes (× 10^9^/L)	2.18 ± 0.46	2.13 ± 0.54	0.8–4.0	> 0.05
Monocytes (× 10^9^/L)	0.62 ± 0.33	0.39 ± 0.43	0.1–0.6	< 0.05
Eosinophils (× 10^9^/L)	0.16 ± 0.24	0.14 ± 0.11	0.05–0.5	> 0.05
Basophils (× 10^9^/L)	0.04 ± 0.11	0.04 ± 0.25	0–0.1	> 0.05

### 3.2 Serum Cytokine and Chemokine Levels

**Figure 1:**
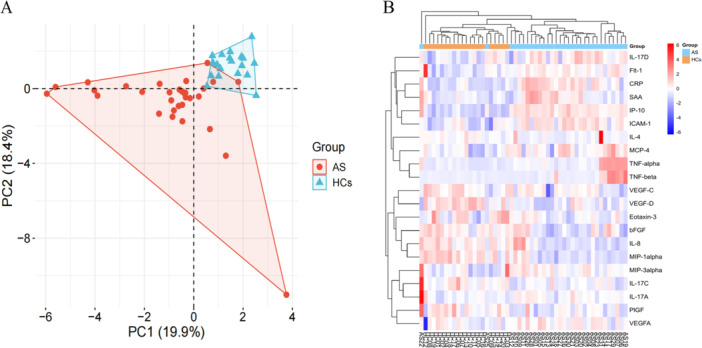
Multivariate statistical analysis of serum cytokines in ankylosing spondylitis patients (AS) and healthy controls (HCs). (A) The scatterplots of principal component analysis (PCA) were based on the serum cytokines data. AS patients and HCs were denoted with blue and red dots, respectively. (B) Heat map of the differential cytokines in AS patients and healthy controls. The row represented the cytokines, and the column represented the individual samples. The deeper the color, the greater the difference in cytokines. Red bands indicated upregulated cytokines, and blue bands indicated downregulated cytokines in the two groups.

To investigate systemic inflammatory alterations in AS, the serum levels of 54 immune mediators were measured in the AS and HC groups. Among them, 50 cytokines and chemokines were measured in more than 75% of the serum samples from both groups and were included in further analysis. The detection rates of the remaining four molecules (IL‐1β, IL‐23, IL‐31, and IL‐1α) were less than 50% and were therefore excluded from further statistical comparisons. The data on these cytokines were imported into the R software to perform PCA. Principal components 1 and 2 accounted for 19.9% and 18.4% of the overall variance of this dataset, respectively (Figure 1A).

**Figure 2:**
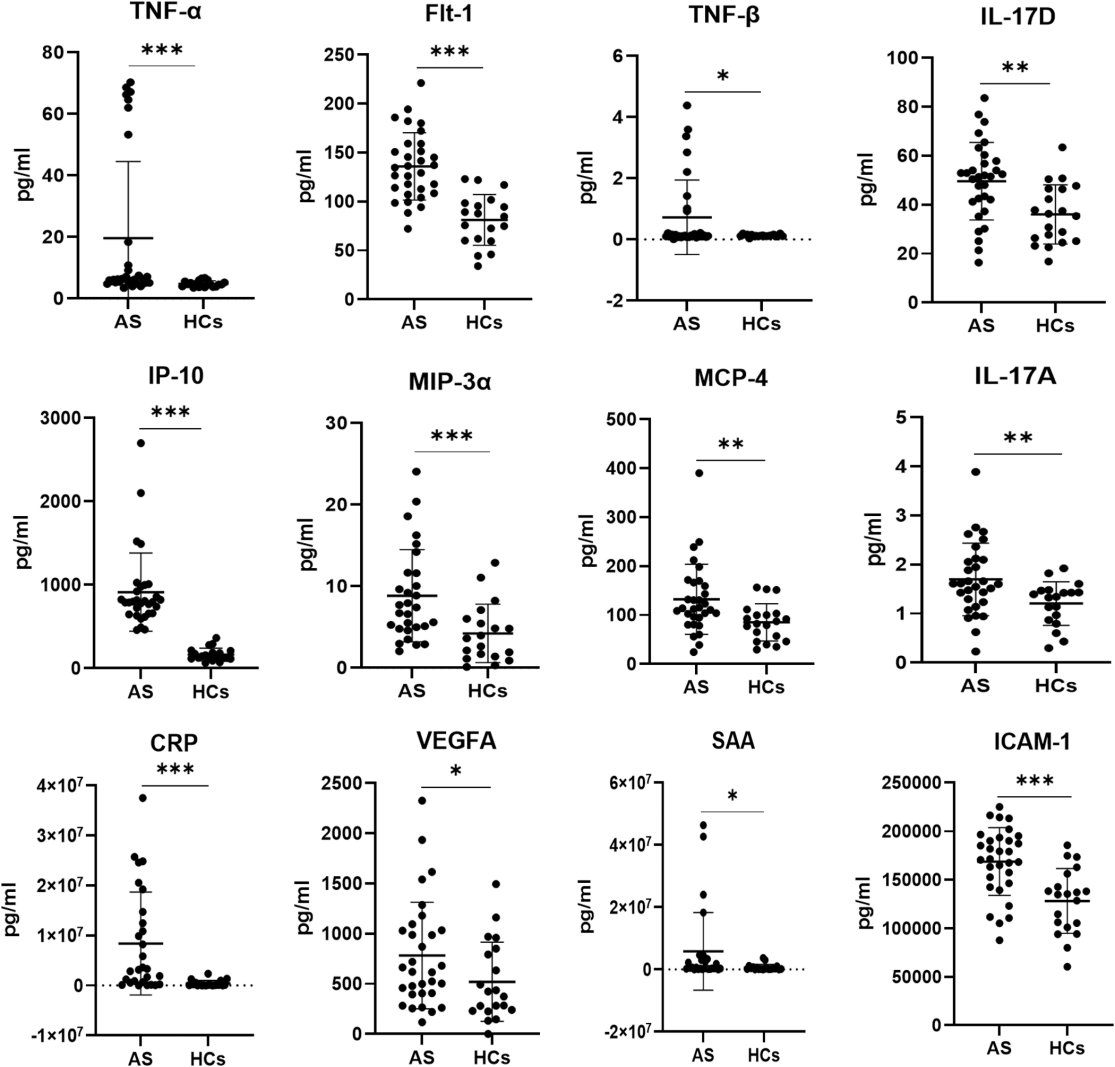
Cytokine/chemokine expressed at higher levels in serum from patients with AS compared to HCs. Mann–Whitney *U*‐test and Student's *t*‐test were carried out to check for statistical significance between groups when required (****p < 0.001, **p < 0.01, *p < 0.05).

In total, 21 cytokines were significantly different between the AS and HC groups. An unsupervised cluster heatmap for 21 cytokines was drawn to understand the expression of the differential cytokines between the AS group and the HCs (Figure 1B). These biomarkers could distinguish most AS patients from HCs. Figure 1A shows that AS and HC groups form two distinct, minimally overlapping clusters, while Figure 1B demonstrates that hierarchical clustering of the 21 differential cytokines segregates most AS patients from HCs, highlighting their discriminatory potential. The levels of 12 cytokines, including TNF‐α, TNF‐β, IL‐17A, VEGFA, ICAM1, SAA, IL‐17D, IP‐10/CXCL10, MIP‐3α/CCL20, sFlt‐1/VEGFR‐1, CRP, and MCP‐4/CCL13, were obviously higher in AS patients (*p* < 0.05; Table 2 and Figure 2). Among these, CRP showed the highest fold change (FC), which was about 40.72‐fold higher in AS patients than in HCs (*p* < 0.0001). Additionally, SAA (FC = 36.67; *p* < 0.0001), TNF‐β (FC = 5.58; *p* = 0.025), and IP‐10/CXCL10 (FC = 5.55; *p* < 0.0001) were significantly higher. Other cytokines in this elevated group presented moderate FCs between 1.32‐fold and 4.36‐fold. In Figure 2, CRP and SAA displayed the largest dynamic range increases, while IP‐10/CXCL10, Flt‐1, and ICAM‐1 also showed marked elevation in AS compared with HCs, reflecting strong systemic inflammatory activation and endothelial involvement.

**Table 2:** Concentrations of cytokines in serum from AS patients and HCs.

	AS (*n* = 31) mean ± SD Median (range)	HCs (*n* = 21) mean ± SD Median (range)	*p* value AS versus HCs	*q* value AS versus HCs	Fold change (FC)
IFN‐γ	9.50 ± 8.58	7.00 ± 2.79	0.296	1	1.36
	7.50 (0.85–45.23)	6.34 (3.29–13.44)			
IL‐10	0.40 ± 0.32	0.39 ± 0.66	0.169	1	1.02
	0.32 (0.06–1.32)	0.26 (0.07–3.17)			
IL‐12p70	0.15 ± 0.13	0.15 ± 0.08	0.345	1	1.00
	0.12 (0.02–0.61)	0.14 (0.07–0.39)			
IL‐13	0.94 ± 0.83	0.89 ± 0.39	0.459	1	1.05
	0.84 (0.07–3.19)	0.89 (0.17–1.59)			
IL‐1β	NA	NA	NA	NA	NA
IL‐2	0.14 ± 0.11	0.17 ± 0.10	0.394	1	0.79
	0.11 (0.01–0.35)	0.15 (0.06–0.44)			
IL‐4	0.01 ± 0.01	0.02 ± 0.01	0.005	0.25	0.65
	0.01 (0.00–0.07)	0.02 (0.01–0.05)			
IL‐6	2.65 ± 3.32	1.06 ± 0.73	0.077	1	2.51
	1.45 (0.11–13.53)	0.90 (0.40–3.64)			
IL‐8	25.27 ± 56.68	56.56 ± 44.60	< 0.0001	0.00055	0.45
	7.88 (2.03–265.03)	48.48 (12.63–205.79)			
TNF‐α	19.62 ± 24.97	4.78 ± 0.99	0.0001	0.005	4.11
	6.26 (3.45–70.31)	4.57 (3.48–6.67)			
Flt‐1	135.94 ± 34.46	81.24 ± 25.99	< 0.0001	C	1.67
	134.67 (72.13–221.17)	84.61 (34.16–123.06)			
PlGF	3.23 ± 1.41	3.86 ± 0.93	0.0003	0.015	0.84
	2.84 (1.81–9.60)	3.71 (2.00–6.25)			
Tie‐2	2054.97 ± 623.41	2239.14 ± 633.72	0.450	1	0.92
	2094.91 (427.01–3171.58)	2275.76 (885.59–3586.14)			
VEGF (plate2)	782.62 ± 531.44	520.67 ± 395.19	0.043	1	1.5
	619.11 (118.50–2326.79)	398.90 (0.31–1494.08)			
VEGF‐C	267.19 ± 108.28	366.72 ± 148.42	0.031	1	0.73
	271.74 (32.17–476.31)	304.67 (116.81–694.61)			
VEGF‐D	1157.69 ± 347.35	1872.14 ± 503.40	< 0.0001	0.00055	0.62
	1095.91 (722.06–2161.77)	1874.64 (1043.63–2742.15)			
bFGF	11.55 ± 11.70	16.38 ± 9.27	0.013	0.65	0.71
	5.80 (0.61–48.01)	16.15 (3.24–37.67)			
IL‐17A/F	2.25 ± 1.86	2.56 ± 1.58	0.474	1	0.88
	1.81 (0.12–7.79)	2.46 (0.66–6.53)			
IL‐17B	8.70 ± 1.88	8.23 ± 1.23	0.506	1	1.06
	8.75 (5.74–14.27)	8.64 (5.74–9.96)			
IL‐17C	6.56 ± 1.27	7.96 ± 1.93	0.001	0.05	0.82
	6.34 (4.60–10.51)	7.43 (6.09–14.58)			
IL‐17D	49.65 ± 15.83	36.03 ± 12.11	0.001	0.05	1.38
	51.53 (16.35–83.66)	35.85 (16.76–63.43)			
IL‐1RA	384.26 ± 139.14	441.26 ± 130.80	0.182	1	0.87
	348.57 (152.04–639.78)	429.57 (274.18–738.57)			
IL‐3	9.45 ± 4.78	8.01 ± 3.29	0.349	1	1.18
	8.60 (1.80–20.01)	7.86 (1.61–14.49)			
IL‐9	1.67 ± 2.63	0.81 ± 0.64	0.260	1	2.07
	0.90 (0.07–13.68)	0.60 (0.14–2.86)			
TSLP	1.87 ± 1.28	1.52 ± 0.46	0.875	1	1.23
	1.57 (0.08–5.97)	1.67 (0.17–2.06)			
Eotaxin	399.39 ± 169.75	376.62 ± 147.62	0.738	1	1.06
	382.68 (171.83–1012.63)	355.37 (154.63–765.65)			
Eotaxin‐3	32.08 ± 9.15	47.95 ± 26.17	0.033	1	0.67
	31.66 (16.64–53.52)	44.36 (13.75–110.77)			
IL‐8(HA)	671.99 ± 440.06	862.14 ± 376.76	0.118	1	0.78
	607.55 (94.85–2034.73)	846.40 (374.93–1525.94)			
IP‐10	911.70 ± 469.12	164.28 ± 76.77	< 0.0001	0.00055	5.55
	792.93 (459.92–2699.43)	142.87 (63.04–364.77)			
MCP‐1	238.32 ± 102.96	242.47 ± 75.23	0.305	1	0.98
	214.39 (111.89–653.39)	242.05 (42.27–370.27)			
MCP‐4	132.20 ± 71.70	85.17 ± 38.11	0.003	0.15	1.55
	114.01 (24.48–390.11)	84.69 (29.27–156.37)			
MDC	1386.63 ± 585.68	1352.17 ± 405.72	0.992	1	1.03
	1330.65 (483.44–2860.00)	1377.39 (752.60–2643.83)			
MIP‐1α	33.72 ± 22.49	86.04 ± 35.89	< 0.0001	0.00055	0.39
	27.30 (9.00–107.72)	88.67 (22.35–164.68)			
MIP‐1β	251.94 ± 89.23	342.05 ± 123.55	0.002	0.1	0.74
	244.29 (96.84–484.29)	329.77 (135.55–610.60)			
TARC	274.46 ± 147.03	217.15 ± 75.61	0.223	1	1.26
	234.23 (86.48–696.15)	216.51 (123.11–406.62)			
IL‐17A Gen.B	2.54 ± 8.99	1.21 ± 0.88	0.088	1	2.10
	0.50 (0.04–44.61)	1.18 (0.01–3.36)			
IL‐21	6.12 ± 22.71	3.00 ± 3.93	0.191	1	2.04
	0.73 (0.04–105.12)	1.30 (0.04–14.10)			
IL‐22	3.13 ± 13.86	0.35 ± 0.25	0.223	1	9.00
	0.48 (0.06–77.74)	0.28 (0.13–0.95)			
IL‐23	NA	NA	NA	NA	NA
IL‐27	1431.61 ± 855.35	1909.86 ± 2130.16	0.534	1	0.75
	1257.90 (348.91–4388.73)	1141.85 (448.76–10297.49)			
IL‐31	NA	NA	NA	NA	NA
MIP‐3α	18.40 ± 52.70	4.22 ± 3.59	0.001	0.05	4.36
	7.56 (2.03–295.89)	3.61 (0.11–12.89)			
GM‐CSF	0.17 ± 0.11	0.20 ± 0.17	0.236	1	0.84
	0.15 (0.05–0.60)	0.16 (0.03–0.87)			
IL‐12/IL‐23p40	126.12 ± 62.26	109.59 ± 36.22	0.305	1	1.15
	117.86 (24.59–344.55)	103.57 (52.24–183.82)			
IL‐15	2.30 ± 0.74	2.31 ± 0.62	0.333	1	1.00
	2.17 (1.39–5.58)	2.35 (0.54–3.36)			
IL‐16	304.41 ± 112.53	274.36 ± 72.32	0.394	1	1.11
	287.58 (135.72–610.33)	272.30 (185.92–469.09)			
IL‐17A	4.18 ± 13.86	1.38 ± 0.88	0.008	0.4	3.04
	1.61 (0.23–78.74)	1.37 (0.30–4.62)			
IL‐1α	NA	NA	NA	NA	NA
IL‐5	0.82 ± 2.39	0.17 ± 0.16	0.313	1	4.79
	0.23 (0.00–11.89)	0.08 (0.02–0.44)			
IL‐7	15.88 ± 7.82	14.16 ± 4.99	0.612	1	1.12
	15.01 (3.43–36.98)	14.70 (4.81–25.48)			
TNF‐β	0.73 ± 1.22	0.13 ± 0.04	0.025	1	5.58
	0.13 (0.01–4.38)	0.12 (0.04–0.20)			
VEGF(plate6)	170.65 ± 123.97	148.21 ± 106.11	0.639	1	1.15
	132.09 (25.21–503.24)	96.30 (0.03–366.34)			
CRP	16167992.66 ± 28121726.14	397075.02 ± 619013.59	< 0.0001	0.00055	40.72
	3615876.31 (14442.04–136158667.77)	101358.35 (17222.15–2343359.07)			
ICAM‐1	168702.92 ± 34857.19	128099.31 ± 33478.60	0.0001	0.005	1.32
	171393.80 (87656.57–225047.91)	134976.67(60301.90–185576.55)			
SAA	23629909.65 ± 63308401.30	644393.62 ± 986344.49	0.048	1	36.67
	606868.44 (15600.03–296313180.50)	271930.05 (13900.46–3681130.66)			
VCAM‐1	251902.82 ± 66863.17	230353.37 ± 58554.93	0.238	1	1.09
	234691.68 (174474.62–432444.07)	225278.06(153524.47–350498.07)			

**Figure 3:**
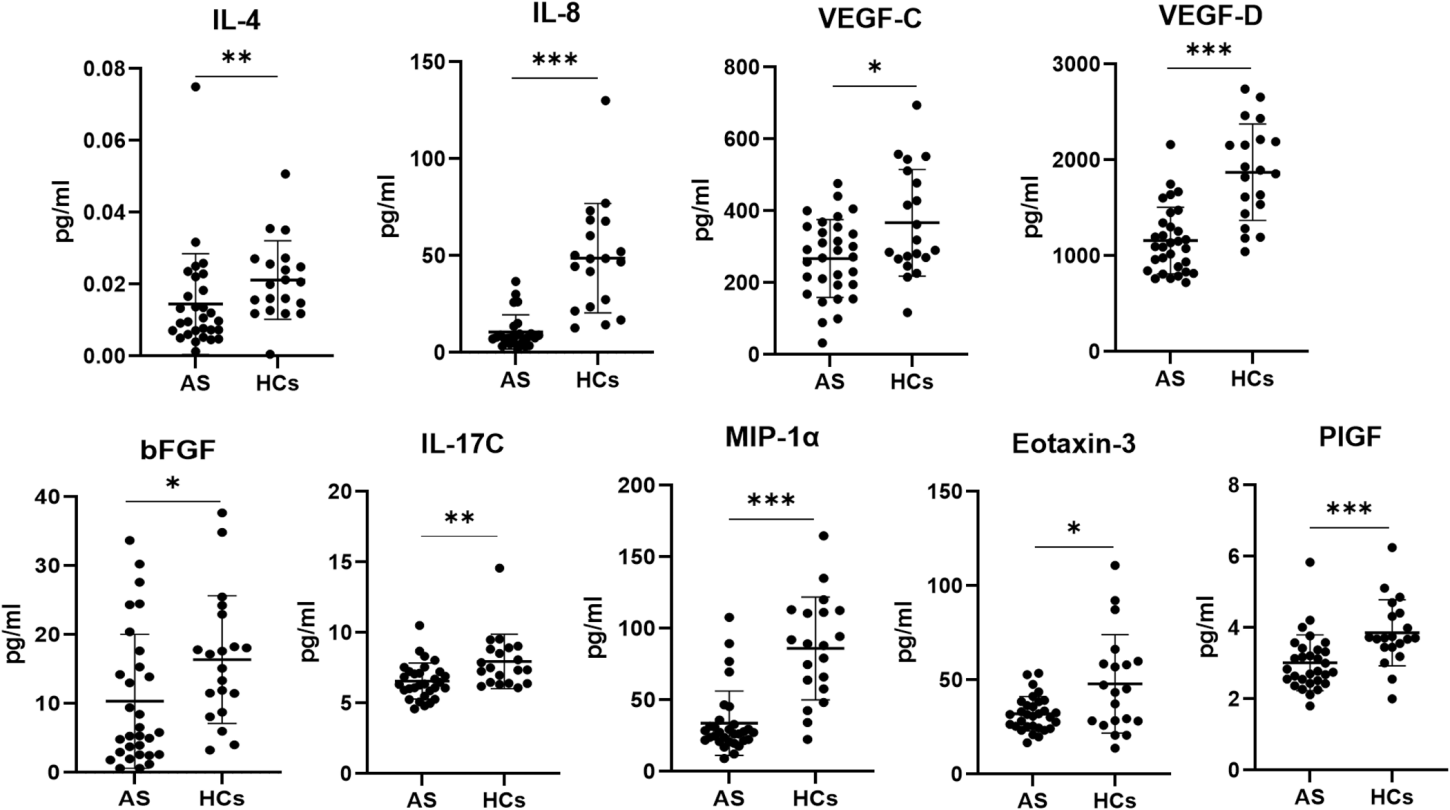
Cytokine/chemokine expressed at lower levels in serum from patients with AS compared to HCs. Mann–Whitney *U*‐test and Student's *t*‐test were carried out to check for statistical significance between groups when required (****p < 0.001, **p < 0.01, *p < 0.05).

In contrast, the serum concentrations of IL‐4, IL‐8, IL‐17C, MIP‐1α/CCL3, eotaxin‐3/CCL26, PlGF, VEGF‐C, bFGF, and VEGF‐D were obviously lower in the AS group than in the HC group (*p* < 0.05; Table 2 and Figure 3). MIP‐1α showed a 0.39‐fold reduction (*p* < 0.0001), whereas IL‐8 showed a 0.45‐fold reduction (*p* < 0.0001). The remaining cytokines exhibited FCs of 0.62–0.84 in the two groups. In Figure 3, VEGF‐D and VEGF‐C showed the most evident reductions in AS patients, accompanied by consistently lower levels of IL‐8, IL‐4, and MIP‐1α, suggesting impaired angiogenic signaling and weakened chemotactic responses in AS.

After the application of the Bonferroni correction for multiple comparisons, eight cytokines (IL‐8, TNF‐α, VEGF‐D, PlGF, IP‐10, MIP‐1α, ICAM1, and CRP) showed significant differences between the AS and HC groups (p < 0.05). However, the remaining 37 analytes were not significantly different (*p* > 0.05; Table 2).

### 3.3 ROC Curve Analysis

**Figure 4:**
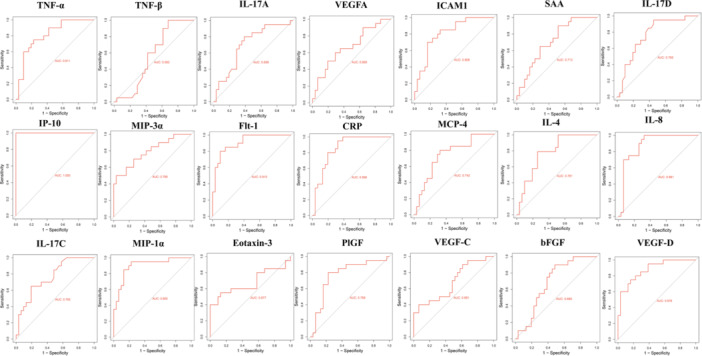
Receiver operating characteristic (ROC) curve analysis of significantly altered cytokines and chemokines in ankylosing spondylitis (AS) patients compared to healthy controls. The analysis includes 12 upregulated and 9 downregulated cytokines. The area under the curve (AUC) and probability (*p*) values are provided. An AUC of 0.50–0.59 indicates no discriminatory ability, 0.60–0.69 suggests poor discrimination, 0.70–0.79 is considered acceptable, and 0.80–0.89 indicates excellent discrimination. AUC values of 0.90 or higher reflect outstanding diagnostic performance.

To analyze whether these cytokines and chemokines can be used to distinguish between AS patients and HCs, ROC curve analysis was performed, with the area under the curve (AUC) used as an evaluation criterion. Based on established standards, an AUC of 0.50–0.59 indicated no diagnostic value, 0.60–0.69 indicated limited or poor discrimination, 0.70–0.79 indicated acceptable diagnostic accuracy, 0.80–0.89 indicated excellent discrimination, and ≥ 0.90 indicated outstanding discriminatory performance [19]. Based on these criteria, our results can be classified into distinct groups (Figure 4). First, TNF‐β (AUC = 0.562) showed no meaningful discriminatory ability. Several cytokines, including bFGF (AUC = 0.685), VEGFA (AUC = 0.669), VEGF‐C (AUC = 0.681), eotaxin‐3 (AUC = 0.677), and IL‐17A (AUC = 0.699), exhibited poor discriminatory ability. Cytokines such as SAA (AUC = 0.713), MCP‐4 (AUC = 0.742), IL‐17C (AUC = 0.765), IL‐17D (AUC = 0.765), PlGF (AUC = 0.769), IL‐4 (AUC = 0.781), and MIP‐3α (AUC = 0.789) showed acceptable diagnostic performance. Cytokines such as TNF‐α (AUC = 0.811), ICAM1 (AUC = 0.808), IL‐8 (AUC = 0.881), and CRP (AUC = 0.856) displayed excellent discriminatory accuracy. IP‐10/CXCL10 (AUC = 1.000), VEGF‐D (AUC = 0.976), MIP‐1α (AUC = 0.900), and sFlt‐1/VEGFR‐1 (AUC = 0.901) exhibited outstanding diagnostic ability (all *p* < 0.001), indicating their strong potential as biomarkers for the diagnosis of AS or disease monitoring. Figure 4 further demonstrates that four cytokines achieved outstanding performance (AUC ≥ 0.90), four showed excellent discrimination, and several others reached acceptable levels, providing a stratified overview of diagnostic value across the panel (Tables 3 and 4).

**Table 3:** Correlation matrix of the increased cytokine concentrations in AS patients and HCs.

	TNF‐a	TNF‐b	Flt‐1	VEGFA	IL‐17D	IP‐10	MCP‐4	MIP‐3a	IL‐17A	CRP	ICAM‐1	SAA
TNF‐a	0											
TNF‐b	0.052534											
Flt‐1	0.016828	0.665378										
VEGFA	0.063609	0.564941	0.978379									
IL17D	0.100805	0.064347	0.024568	0.077195								
IP‐10	0.001939	0.763084	0.000007	0.043430	0.003956							
MCP‐4	0.008338	0.143438	0.006179	0.169225	0.633627	0.024301						
MIP‐3a	0.000611	0.342658	0.003719	0.285986	0.947480	0.000084	0.014021					
IL‐17A	0.027042	0.377822	0.010331	0.329353	0.330568	0.004358	0.404258	0.025912				
CRP	0.008348	0.326958	0.003141	0.001689	0.059516	0.000005	0.076560	0.000064	0.003225			
ICAM‐1	0.008845	0.997485	0.005029	0.038517	0.077973	0.000008	0.034094	0.002436	0.159367	0.000001		
SAA	0.487831	0.599244	0.010429	0.037765	0.078870	0.000403	0.979384	0.012789	0.012537	0.000000	0.025401	0

*Note:* Spearman's correlation was calculated for every pair of datasets. *p* values are shown after the Bonferroni correction.

**Table 4:** Correlation matrix of the decreased cytokine concentrations in AS patients and HCs.

	IL‐4	IL‐8	PlGF	VEGF‐C	VEGF‐D	bFGF	IL‐17C	Eotaxin‐3	MIP‐1a
IL‐4	0								
IL‐8	0.025243								
PlGF	0.378847	0.108937							
VEGF‐C	0.892434	0.193994	0.119596						
VEGF‐D	0.101686	0.014587	0.113199	0.014840					
bFGF	0.150359	0.000041	0.080839	0.037455	0.668395				
IL‐17C	0.675566	0.197171	0.036211	0.218013	0.102245	0.384586			
Eotaxin‐3	0.483480	0.062238	0.016220	0.007720	0.640770	0.048415	0.374016		
MIP‐1a	0.000320	0.000000	0.012322	0.022917	0.003745	0.000015	0.088535	0.079797	0

*Note:* Spearman's correlation was calculated for every pair of datasets. *p* values are shown after the Bonferroni correction.

### 3.4 Validation of IP‐10/CXCL10 and VEGF‐D Expression in SKG Mice via qPCR

**Figure 5:**
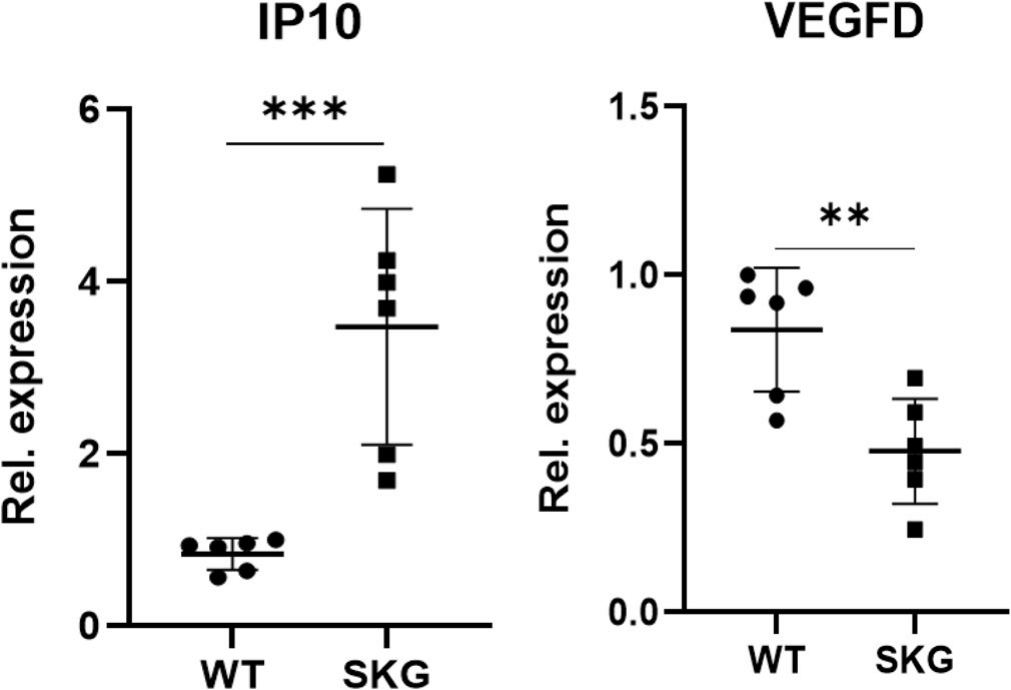
RT‐PCR validation of IP‐10/CXCL10 and VEGF‐D expression in PBMCs from SKG mice. Quantitative real‐time RT‐PCR analysis showed significantly increased expression of IP‐10/CXCL10 and decreased expression of VEGF‐D in peripheral blood mononuclear cells (PBMCs) from SKG mice compared to wild‐type controls (*n* = 6 per group; *p* < 0.01, unpaired *t*‐test). Data are presented as mean ± SEM.

To validate the clinical findings, we examined the mRNA expression levels of IP‐10/CXCL10 and VEGF‐D in PBMCs isolated from SKG mice by conducting qPCR. SKG mice are frequently used as animal models because they spontaneously develop spondyloarthritis‐like features, which makes them suitable for investigating AS‐related immune mechanisms [20]. As shown in Figure 5, IP‐10/CXCL10 was significantly upregulated, whereas VEGF‐D was significantly downregulated in SKG mice compared to their wild‐type counterparts (*p* < 0.01). These results were consistent with the protein‐level alterations found in the serum of AS patients; therefore, our findings support the translational relevance of these cytokines across species. In Figure 5, the opposite trends of increased IP‐10/CXCL10 and decreased VEGF‐D in SKG mice parallel the alterations observed in human serum, reinforcing their consistency across species. These results reinforce the potential of IP‐10/CXCL10 and VEGF‐D as robust biomarkers associated with AS‐related immune dysregulation.

### 3.5 Spearman's Rank Correlation Analysis

Correlation analyses were adopted for examining interactions among cytokines and chemokines that were significantly altered in AS patients (Tables S1 and S2). Among the cytokines elevated in AS patients, IP‐10/CXCL10 showed a significant correlation with TNF‐β (*p* = 0.000007), whereas ICAM1 was significantly correlated with sFlt‐1/VEGFR‐1 (*p* < 0.0001). Additionally, TNF‐α was significantly correlated with several inflammatory mediators, including CRP (*p* = 0.0038), MIP‐3α/CCL20 (*p* = 0.000611), MCP‐4 (*p* = 0.0083), and IP‐10/CXCL10 (*p* = 0.0019), suggesting that the inflammatory pathways were coordinated. Among the cytokines with reduced serum levels, IL‐4 was significantly related to VEGF‐C (*p* = 0.000041), IL‐17C (*p* = 0.00032), and MIP‐1α/CCL3 (*p* = 0.00032), indicating that interactions among anti‐inflammatory and angiogenesis‐related cytokines may occur in AS pathogenesis. These associations highlight complex networks of immune activation, chemokine‐driven inflammation, and angiogenesis dysregulation contributing to the mechanisms underlying AS.

## 4 Discussion

AS is an autoimmune inflammatory condition mostly involving the spine and sacroiliac joints, and its typical features are chronic inflammation, structural damage, and progressive disability. This study comprehensively investigated the serum profiles of 54 cytokines and chemokines in AS patients compared to HCs. Our results revealed significantly higher serum levels of several cytokines and chemokines, including TNF‐α, TNF‐β, IL‐17A, IL‐17D, VEGFA, ICAM1, IP‐10/CXCL10, MIP‐3α/CCL20, sFlt‐1/VEGFR‐1, CRP, MCP‐4/CCL13, and SAA, whereas the serum levels of IL‐4, IL‐8, IL‐17C, MIP‐1α/CCL3, eotaxin‐3/CCL26, PlGF, VEGF‐C, VEGF‐D, and bFGF were significantly lower. Additionally, by conducting RT‐PCR using PBMCs from SKG mice, we found higher expression of IP‐10/CXCL10 and lower expression of VEGF‐D at the transcriptional level, which was consistent with clinical observations. These findings highlight the complexity of immune dysregulation and angiogenic imbalance in AS, revealing potential biomarkers and therapeutic targets for future research.

Several studies have found that cytokines serve as central mediators of the pathogenesis of AS, with the TNF‐α and IL‐23/IL‐17 axes recognized as essential inflammatory pathways [18, 21]. Among these cytokines, the level of TNF‐α, a classical pro‐inflammatory cytokine, is elevated in AS, making TNF‐α inhibitors the most effective first‐line biological therapeutic agents recommended by both the EULAR and the guidelines [22, 23]. Our findings align with these reports, confirming significantly elevated TNF‐α levels in AS patients. Moreover, we found an increase in serum TNF‐β levels in AS patients, a finding previously reported in AS patients. TNF‐β induces inflammatory responses in human chondrocytes [24]; therefore, it may be involved in the inflammatory joint damage characteristic of AS, and further studies are needed to confirm this speculation. However, further studies, including mechanistic experiments, should be performed to verify the specific role of TNF‐β in AS.

We also found distinct expression patterns of IL‐17 family cytokines in the serum of AS patients. The IL‐17A and IL‐17D levels significantly increased, whereas the IL‐17C levels significantly reduced. The elevated IL‐17A levels observed in our study align with previous findings, confirming the central role of Th17‐mediated inflammatory responses in AS pathogenesis [20]. However, the increase in IL‐17D has not been widely reported in AS. This cytokine, which acts through alternative receptor pathways, could contribute to innate immune activation and inflammatory cell recruitment, as previously suggested in studies of other autoimmune diseases [25]. In contrast, the reduction in the level of IL‐17C, a mediator involved predominantly in mucosal barrier immunity and innate defense [26], may suggest compromised barrier function or dysregulation of innate immunity in AS. These differential cytokine profiles indicate a complex interplay among members of the IL‐17 family, suggesting that classical Th17‐driven inflammation and dysregulated innate immunity are related to AS, highlighting the need for further studies on their specific roles and interactions.

Chemokines play key roles in immune regulation by mediating the recruitment of leukocytes and modulating inflammatory responses in autoimmune diseases. We found significantly higher serum IP‐10/CXCL10, MIP‐3α/CCL20, and MCP‐4/CCL13 levels among AS patients, whereas MIP‐1α/CCL3 and eotaxin‐3/CCL26 levels were significantly lower. Among these, IP‐10/CXCL10, a chemokine that mediates the recruitment of Th1 cells, is associated with chronic inflammatory diseases [27]. Wang et al. reported an increase in serum IP‐10 levels among AS patients, which was positively associated with disease activity indicators, such as CRP and ESR, indicating that serum IP‐10 may be regarded as a candidate biomarker for disease activity [28]. Our ROC analysis confirmed that IP‐10 had the highest diagnostic accuracy, indicating that it may be a strong disease biomarker. MCP‐4/CCL13 levels increase under some chronic inflammatory conditions, such as multiple sclerosis and RA [29, 30]. MIP‐3α/CCL20, another significantly elevated chemokine in our study, is crucial for recruiting CCR6^+^ Th17 cells to inflammatory sites and is involved in various autoimmune diseases [31]. An increase in serum levels of MIP‐3α/CCL20 may suggest enhanced Th17‐driven inflammation in AS, suggesting that the IL‐17 axis is vital for this disease. In contrast, our data suggested a significant reduction in the levels of MIP‐1α/CCL3 and eotaxin‐3/CCL26. Previous studies reported high serum MIP‐1α levels in AS and RA patients, which decreased after administering anti‐TNF‐α treatment, which is different from our findings [32]. This discrepancy may reflect differences in disease stage, prior treatments, or patient cohort characteristics. Eotaxin‐3/CCL26, which is involved primarily in eosinophil recruitment and allergic responses [33, 34], has been extensively investigated in AS pathogenesis, and our novel finding of its reduction highlights the role of altered eosinophil‐associated immune responses. Comparative studies involving multiple inflammatory disorders are needed to evaluate the specificity and diagnostic utility of these chemokines for AS.

The serum concentrations of several VEGF family members, notably VEGF‐C and VEGF‐D, were lower, whereas VEGFA and sFlt‐1/VEGFR‐1 were higher in AS patients. The members of the VEGF family have important effects on angiogenesis and lymphangiogenesis, processes related to autoimmune diseases. High levels of VEGF‐A are associated with pathological angiogenesis and chronic inflammation in arthritic conditions such as RA and AS [35, 36], suggesting its role in driving persistent inflammatory processes. In support of these findings, an AS mouse model of proteoglycan‐induced arthritis (PGIA) demonstrated that intra‐articular administration of sFlt‐1 reduced the severity of arthritis and macrophage infiltration by decreasing VEGFR‐2‐mediated vascular permeability [37]. These findings align with our observed increase in sFlt‐1 levels. Additionally, correlation analysis suggested a significant positive relationship between VEGF‐A and sFlt‐1, suggesting a tightly regulated angiogenic response, with sFlt‐1 acting as a compensatory mechanism to counterbalance excessive VEGF‐A‐driven vascular activation. In contrast, a decrease in serum VEGF‐C and VEGF‐D levels, which differs from previous reports of locally increased VEGF‐C expression in arthritic synovium but aligns with reported minimal expression of synovial VEGF‐D in AS and RA [38, 39], indicates potential impairment of lymphangiogenic signaling or altered vascular permeability. These results revealed complex and distinct patterns of VEGF family dysregulation in AS, characterized by simultaneous activation of angiogenic pathways (VEGF‐A and sFlt‐1) and suppression of lymphangiogenic mediators (VEGF‐C and VEGF‐D). Longitudinal studies are needed to elucidate the temporal and causal relationships between the above angiogenic mediators and the progression of AS.

This study had several limitations. Its small sample size probably limited its statistical power and generalizability. A further limitation is that the sample size was determined based on feasibility rather than a priori power calculation, and future studies with larger cohorts and predefined sample size estimation will be necessary to validate these findings. Additionally, this study did not evaluate cytokine specificity against other inflammatory diseases or compare cytokine‐based diagnostic performance directly with established clinical diagnostic standards. Longitudinal studies with larger cohorts, including multiple inflammatory disease controls, are needed to validate these preliminary findings.

To summarize, this study characterized the serum cytokine and chemokine profiles of AS patients, revealing dysregulated inflammatory and angiogenic pathways. IP‐10/CXCL10 and VEGF‐D showed high diagnostic accuracy and potential as biomarkers. RT‐PCR validation of PBMCs from SKG mice confirmed their expression patterns, which were consistent with the clinical findings. New and unreported cytokines and chemokines related to AS were also identified. While promising, the specificity and clinical applicability of these markers need to be determined in larger human and animal cohorts. These results provided novel information for diagnosing AS and developing therapeutic strategies. Future research should focus on understanding the longitudinal effects of these markers over time and their potential role in disease progression.

## Author Contributions

Bo Gong and Jing Zhu were in charge of study conceptualization. Huan Li, Wei Su, Ying Lv, Jialing Xiao, Xiaoxin Guo, Kai Dong, and Chengzi Gan collected samples. Bo Gong and Jing Zhu were responsible for data analysis. Huan Li and Wei Su wrote the manuscript. Ting Wang and Mingze Li contributed to scientific content analysis and manuscript revision. The authors agreed to this manuscript.

## Ethics Statement

This study gained approval from the Human Ethics Committee of Sichuan Provincial People's Hospital, Affiliated Hospital of the University of Electronic Science and Technology of China (Approval No. 2020. NO. 7). All procedures were carried out following the Declaration of Helsinki.

## Consent

Every participant provided informed consent before their inclusion in this study.

## Conflicts of Interest

The authors declare no conflicts of interest.

## Supporting information


**Supporting Table 1**: Correlation matrix of the increased cytokine concentrations in AS patients and HCs.

**Supporting Table 2**: Correlation matrix of the decreased cytokine concentrations in AS patients and HCs.

## Data Availability

Data supporting our results can be obtained from the corresponding author upon request.
