# Supplementary material for: Serum Levels of 54 Cytokines and Chemokines Reveal Distinct Inflammatory Signatures in Ankylosing Spondylitis
Source: Immun Inflamm Dis. 2025 Oct 15;13(10):e70276. doi: 10.1002/iid3.70276 (PMC12521873; doi:10.1002/iid3.70276)
Supplement: Supplementary file 1 — Supporting Table 1: Correlation matrix of the increased cytokine concentrations in AS patients and HCs. [file IID3-13-e70276-s001.docx]

**Supplemental Table 1**. Correlation matrix of the increased cytokine concentrations in AS patients and HCs.

|  | TNF-a | TNF-b | Flt-1 | VEGFA | IL-17D | IP-10 | MCP-4 | MIP-3a | IL-17A | CRP | ICAM-1 | SAA |
| --- | --- | --- | --- | --- | --- | --- | --- | --- | --- | --- | --- | --- |
| TNF-a | 0 |  |  |  |  |  |  |  |  |  |  |  |
| TNF-b | 0.052534 |  |  |  |  |  |  |  |  |  |  |  |
| Flt-1 | 0.016828 | 0.665378 |  |  |  |  |  |  |  |  |  |  |
| VEGFA | 0.063609 | 0.564941 | 0.978379 |  |  |  |  |  |  |  |  |  |
| IL-17D | 0.100805 | 0.064347 | 0.024568 | 0.077195 |  |  |  |  |  |  |  |  |
| IP-10 | 0.001939 | 0.763084 | 0.000007 | 0.043430 | 0.003956 |  |  |  |  |  |  |  |
| MCP-4 | 0.008338 | 0.143438 | 0.006179 | 0.169225 | 0.633627 | 0.024301 |  |  |  |  |  |  |
| MIP-3a | 0.000611 | 0.342658 | 0.003719 | 0.285986 | 0.947480 | 0.000084 | 0.014021 |  |  |  |  |  |
| IL-17A | 0.027042 | 0.377822 | 0.010331 | 0.329353 | 0.330568 | 0.004358 | 0.404258 | 0.025912 |  |  |  |  |
| CRP | 0.008348 | 0.326958 | 0.003141 | 0.001689 | 0.059516 | 0.000005 | 0.076560 | 0.000064 | 0.003225 |  |  |  |
| ICAM-1 | 0.008845 | 0.997485 | 0.005029 | 0.038517 | 0.077973 | 0.000008 | 0.034094 | 0.002436 | 0.159367 | 0.000001 |  |  |
| SAA  Spearman’s correlation was calculated for every pair of data set. P-values are shown after the Bonferroni correction. | 0.487831 | 0.599244 | 0.010429 | 0.037765 | 0.078870 | 0.000403 | 0.979384 | 0.012789 | 0.012537 | 0.000000 | 0.025401 | 0 |
