# Supplementary material for: Serum Levels of 54 Cytokines and Chemokines Reveal Distinct Inflammatory Signatures in Ankylosing Spondylitis
Source: Immun Inflamm Dis. 2025 Oct 15;13(10):e70276. doi: 10.1002/iid3.70276 (PMC12521873; doi:10.1002/iid3.70276)
Supplement: Supplementary file 2 — Supporting Table 2: Correlation matrix of the decreased cytokine concentrations in AS patients and HCs. [file IID3-13-e70276-s002.docx]

**Supplemental Table 2**. Correlation matrix of the decreased cytokine concentrations in AS patients and HCs.

|  | IL-4 | IL-8 | PlGF | VEGF-C | VEGF-D | bFGF | IL-17C | Eotaxin-3 | MIP-1a |
| --- | --- | --- | --- | --- | --- | --- | --- | --- | --- |
| IL-4 | 0 |  |  |  |  |  |  |  |  |
| IL-8 | 0.025243 |  |  |  |  |  |  |  |  |
| PlGF | 0.378847 | 0.108937 |  |  |  |  |  |  |  |
| VEGF-C | 0.892434 | 0.193994 | 0.119596 |  |  |  |  |  |  |
| VEGF-D | 0.101686 | 0.014587 | 0.113199 | 0.014840 |  |  |  |  |  |
| bFGF | 0.150359 | 0.000041 | 0.080839 | 0.037455 | 0.668395 |  |  |  |  |
| IL-17C | 0.675566 | 0.197171 | 0.036211 | 0.218013 | 0.102245 | 0.384586 |  |  |  |
| Eotaxin-3 | 0.483480 | 0.062238 | 0.016220 | 0.007720 | 0.640770 | 0.048415 | 0.374016 |  |  |
| MIP-1a  Spearman’s correlation was calculated for every pair of data set. P-values are shown after the Bonferroni correction. | 0.000320 | 0.000000 | 0.012322 | 0.022917 | 0.003745 | 0.000015 | 0.088535 | 0.079797 | 0 |
